# Use of Epidermal Growth Factor Receptor Mutation Analysis in Patients with Advanced Non-Small-Cell Lung Cancer to Determine Erlotinib Use as First-Line Therapy

**DOI:** 10.1371/currents.RRN1245

**Published:** 2011-06-21

**Authors:** Naoko Ishibe, Josh Carlson, Scott David Ramsey, Andrew Freedman, Sheri Schully

**Affiliations:** ^*^Program Director, CTEB, DCCPS, National Cancer Institute, NIH, Bethesda, MD; ^†^Institute of Public Health Genetics, School of Public Health and Community Medicine, University of Washington, Seattle, WA; ^‡^Physician, Health Services Researcher; ^§^Genomic Pharmacoepidemiology, US National Cancer Institute and ^¶^Health Sciences Administrator, NCI, Bethesda, MD

## Abstract

Lung cancer is the second most common cancer and the leading cause of cancer-related deaths in the United States. Moreover, advanced non-small-cell lung cancer (NSCLC) is considered an incurable disease and current treatment approaches provide marginal improvement in overall survival at the expense of substantial morbidity and mortality, highlighting the need for new, less toxic treatment approaches. Tyrosine kinase inhibitors, such as erlotinib (Tarceva®), have been developed and approved as maintenance, second- and third-line treatment options in unselected advanced NSCLC patients (2, 15). However, subgroup analyses from the initial clinical trials consistently showed that patients with epidermal growth factor receptor (EGFR) mutations who received erlotinib had higher rates of response and better progression-free and overall survival, leading to clinical trials specifically focused on the use of tyrosine kinase inhibitors as first-line therapy in these patients. We examined the published literature on the analytic validity, clinical validity, and clinical utility of EGFR mutational testing in guiding first-line therapy use of erlotinib to treat advanced NSCLC and we briefly summarized the current lung cancer screening guidelines. The primary goal was to provide a basic overview of EGFR mutational testing and use of erlotinib as first-line therapy and identify gaps in knowledge and evidence that affect the recommendation and adoption of the test in advanced NSCLC treatment management strategies.

**Clinical Scenario**

Testing patients with advanced non-small-cell lung cancer (NSCLC) for epidermal growth factor receptor (*EGFR*) mutation status may help determine who is more likely to respond to the tyrosine kinase inhibitor erlotinib. Recent data suggest that patients with *EGFR* mutations are more responsive to treatment with erlotinib and gefitinib than those without mutations, which may make it possible for doctors and patients to tailor therapy at diagnosis. Nevertheless, several uncertainties remain regarding *EGFR*, including: (1) the prevalence of this mutation among all patients with NSCLC; (2) the need for a second biopsy, given that testing for *EGFR* requires volumes of tumor that are often beyond that obtained for diagnosis; and (3) the optimal *EGFR* testing strategy (i.e., test all patients or confine testing to patients with correlated clinical characteristics.  Those issues may strongly influence the effectiveness and cost-effectiveness of *EGFR* testing as a management strategy for patients with NSCLC.

**Test Description**

*EGFR* mutation analysis detects acquired mutations in the *EGFR* gene in tumor specimens of patients with NSCLC.  There are various polymerase chain reaction (PCR)-methods that can detect the mutations, as well as through the use of direct sequencing, which is currently considered the gold standard. 

**Public Health Importance**

Lung cancer is the second most commonly diagnosed cancer in the United States, with NSCLC accounting for approximately 80% of all lung cancer cases.  Lung cancer is the leading cause of cancer-related deaths in both men and women in the U.S. [1]. Current first-line therapy of advanced NSCLC is platinum-based doublet chemotherapy and is usually limited to four to six cycles. Tyrosine kinase inhibitors, such as erlotinib, have been developed and approved for use in maintenance (i.e., after platinum-based first-line treatment without progression) and second-line treatment of NSCLC [2] [3].  Approximately 10-20% of NSCLC tumors have a somatic mutation in *EGFR*, but prevalence varies among patients with different phenotypes (e.g., women, Asians, non-smokers versus smokers, cancer histology). 

**Published Reviews, Recommendations and Guidelines**

**Systematic evidence reviews**

      Blue Cross Blue Shield Association, Technology Evaluation Center (BCBS TEC) – BCBS TEC concluded that there is sufficient evidence to conclude clinical validity or utility of *EGFR* mutation testing to predict response to erlotinib and to guide treatment decisions in patients with NSCLC [4]. 

**Recommendations by independent group**

      None identified. 

**Guidelines by professional groups (in order by year of publication)**

- American Society of Clinical Oncology (ASCO) – recommended that patients with NSCLC who are being considered for first-line therapy with an EGFR tyrosine kinase inhibitor (TKI) should have their tumors tested for *EGFR* mutations* *to determine whether EGFR TKI or chemotherapy is administered as first-line therapy [5].
- European Medicines Agency – currently only recommends the use of erlotinib for second-line therapy (in NSCLC patients with *EGFR*-positive tumors); Roche has applied to include its use in first-line treatment in patients with advanced NSCLC with *EGFR* mutations [6].  Gefitinib, a similar tyrosine kinase inhibitor, is approved in the first-line treatment in patients with advanced NSCLC with *EGFR* mutations.
- Expert Panel Meeting of the Italian Association of Thoracic Oncology – recommended that *EGFR* mutational analysis, at present, is not recommended in all patients [7].  It should be performed in subgroups of patients characterized by higher prevalence of *EGFR* mutations (e.g., Asians, never smokers, women, adenocarcinoma).  The group recommended that in patients with *EGFR *mutations, an EGFR TKI may be considered as first-line treatment.
- In a recent consensus meeting of Canadian medical oncologists in March 2010, the group recommended:

- “….that diagnostic lung cancer samples of patients with non-small cell lung cancer be routinely tested for activating mutations of the
- *EGFR*
- .  Given the available clinical data, this testing should be limited to patients with advanced NSCLC and non-squamous histology…..Mutation testing is most relevant to treatment decisions in the first-line therapy setting and may also be relevant whenever a choice between chemotherapy and EGFR TKIs is to be made.” 
- [8]

      National Comprehensive Cancer Network (NCCN) – In the V1.2011 Guidelines [9], NCCN recommended *EGFR* mutation testing for the following lung cancer histologies: adenocarcinoma, large cell, and NSCLC, not otherwise specified.  They also recommend the use of erlotinib as first-line therapy for patients who have an *EGFR *mutation. 

A summary of all mentioned recommendations and guidelines appear in Table 1 below.

**Table 1**.  **Recommendations by Organization** ^**1**^ **as to the Utility of *EGFR* Mutation Testing for Erlotinib Therapy in NSCLC.**

**Table:** 

**Organization**	**Evaluated**	**Treatment** **First-line Second-line**	
BCBS TEC	Yes	Yes	Yes
ASCO	Yes	Recommended in a subset of patients	Yes
NCCN	Yes	Recommended in a subset of patients	Yes
European Medicines Agency^2^	Yes	No	Yes
Italian Association of Thoracic Oncology	Yes	Recommended in a subset of patients	Recommended in subset of patients

**Evidence Overview**

***Analytic Validity*: **test accuracy and reliability in detection of *EGFR *mutations using PCR-based methods (analytic sensitivity and specificity).    

      Canadian Agency for Drugs and Technologies in Health (CADTH) recently conducted a review of the literature (11 observational studies) to assess the diagnostic performance of PCR-based methods to detect the presence of *EGFR* mutations in patients with advanced NSCLC [8].  The report concluded that PCR-based methods can identify mutations in *EGFR* with a similar sensitivity to that of direct sequencing. 

      Molina-Vila *et al.* [10] evaluated a PCR-based method that requires two rounds of PCR amplification compared with DNA sequencing.  The results from the PCR method were identical to those obtained from sequencing in 86% of cases for exon 19 and 78% for exon 21. 

      Hoshi *et al.* [11] used the Smart Amplification Process (SMAP) and direct sequencing to rapidly detect mutation in *EGFR*.  Nine mutations were identified using both methods.  Although sensitivity and specificity were not reported, SMAP was able to detect an additional mutation that was not identified using sequencing, suggesting that SMAP may be able to better detect mutations in samples with low DNA (i.e., limited specimen material^3^) which is a limitation of direct sequencing.  It has also been noted that in some patients, re-biopsying tumors would be necessary to obtain enough sample to test for *EGFR*. 

      To date, no mutation analysis test has been approved by the FDA. 

***Clinical Validity*: **strength of relationship between test result and patient outcomes (predictive and prognostic value).

- Jackman *et al.* [12] reported that chemotherapy-naive patients who had *EGFR*-mutation positive NSCLC had statistically significant better outcomes than those with wild-type *EGFR* when treated with a tyrosine kinase inhibitor, such as erlotinib. The authors also reported that *EGFR* genotype was more effective than clinical characteristics at selecting appropriate patients for consideration of first-line therapy with an EGFR-TKI.  In the group with 3 or 4 clinical predictors – race, gender, smoking status, and tumor histology – *EGFR* mutation status was further able to divide the 59 patients into two groups and predict the patients who would benefit from erlotinib (time-to-progression [TTP]=12.9 months in *EGFR*-mutation positive patients versus TTP=1.8 months in *EGFR*-mutation negative patients, p<0.0001).  Within the 164 patients with zero to two clinical predictors, *EGFR* mutation status provided additional information to better determine who would benefit from erlotinib treatment (TTP=10.8 months in *EGFR*-mutation positive patients versus TTP=2.5 months, p<0.0001).  
- Results from the Phase III OPTIMAL trial [13] found that in 165 patients with *EGFR*-mutation positive advanced NSCLC first-line treatment with erlotinib was statistically significantly more effective than standard chemotherapy.  The investigators reported that median progression-free survival was 13.1 months in the erlotinib arm compared to 4.6 months in the chemotherapy arm.  
- The Phase III European Randomized Trial of Tarceva versus Chemotherpay (EURTAC) found that erlotinib extended progression-free survival in Caucasian patients with *EGFR*-mutation positive advanced NSCLC compared with standard chemotherapy.  The authors reported that median progression-free survival was 9.4 months in the erlotinib arm compared to 5.2 months in the chemotherapy arm (p<0.0001) [14].

***Clinical Utility*: net benefit of test in improving health outcomes.**  

      Currently no prospective, randomized clinical trial has specifically looked at how *EGFR*-directed therapy affects patient outcomes compared to standard management of all newly diagnosed patients with NSCLC.  

      Preliminary modeling analysis by the Center for Comparative Effectiveness in Genomic Medicine (unpublished) suggests that *EGFR* mutation testing is moderately cost-effective. 

**Limitations**

      Different studies used different PCR-based methods to detect *EGFR* mutations which may limit the comparability of the results across studies.   

**Conclusions**

As noted earlier, lung cancer is the leading cause of cancer-related deaths, with an estimated 157,300 deaths in the US in 2010 [15].  Moreover, advanced NSCLC is considered an incurable disease and current treatment approaches provide marginal improvement in overall survival at the expense of substantial morbidity and mortality, highlighting the need for new, less toxic treatment approaches.  

Tyrosine kinase inhibitors, such as erlotinib, have been developed and approved as maintenance, second- and third-line treatment options in unselected advanced NSCLC patients [2] [16]. However, subgroup analyses from the initial clinical trials consistently showed that patients with *EGFR* mutations and correlated clinical and histological characteristics who received erlotinib had higher rates of response and better progression-free and overall survival, leading to clinical trials specifically focused on the use of tyrosine kinase inhibitors as first-line therapy in these patients.  

Overall, the available evidence indicates that upfront *EGFR* mutation testing for patients with advanced NSCLC can help guide treatment decisions (i.e., *EGFR* mutation status can identify NSCLC patients who are more likely to respond to erlotinib, whereas those with wild-type tumors are unlikely to respond to erlotinib and alternative treatment should be considered). However, there are remaining gaps in the evidence base that make strong conclusions about the clinical utility premature: (1) there are currently no data from prospective randomized clinical trials comparing *EGFR *mutation testing to usual care;(2) there is a lack of data as to whether *EGFR-TKI’s *provide an overall survival advantage when compared with platinum-based treatment in patients with *EGFR *mutations; and (3) the cost-effectiveness of erlotinib as first-line or maintenance therapy, considering the high cost of a monthly supply^4^, has not been adequately assessed.

**Links**

Last updated: June 13, 2011

**Acknowledgments**

The authors would like to thank Dr. Muin Khoury of the Centers for Disease Control and Prevention and Dr. David Veenstra of the University of Washington for their invaluable input and guidance on the content.  The authors also acknowledge the contributions of Ms. Camillo Benedicto and Ms. Kelly Bennett of the National Cancer InstiFunding was provided in part by CANCERGEN: Comparative Effectiveness

**Funding**

****

This study was funded by CANCERGEN (Comparative Effective Research in Cancer Genomics) through the American Recovery and Reinvestment Act of 2009 by the National Cancer Institute, National Institutes of Health under Agency Award # RC2 CA148570 (Principle Investigator: Scott D. Ramsey).

**Competing interests**

Josh Carlson has been a consultant to Genentech, Inc. and Biodesix, Inc.  All other authors have declared no competing interests exist.

**Disclaimers**

The findings and conclusions are those of the authors and do not necessarily represent the views of the National Institutes of Health (NIH).  The information provided in this manuscript does not constitute an endorsement of EGFR mutation testing by NIH nor the Department of Health and Human Services of the U.S. government.    

**REFERENCES**

[1] National Cancer Institute, 2009.  A Snapshot of Lung Cancer. Accessed November 26, 2010.

[2]  Cappuzzo F, Ciuleanu T, Stelmakh L, et al.  Erlotinib as maintenance treatment in advanced non-small-cell lung cancer: a multicentre, randomized, placebo-controlled phase 3 study.  Lancet Oncol 11:521-529, 2010.  

[3]  U.S. Food and Drug Administration, 2010. http://www.fda.gov/AboutFDA/CentersOffices/CDER/ucm209058.htm. Accessed December 21, 2010.

[4]  Blue Cross Blue Shield.  Epidermal Growth Factor Receptor Mutations and Tyrosine Kinase Inhibitor Therapy in Advanced Non-Small Cell Lung Cancer.  http://www.bcbs.com/blueresources/tec/press/epidermal-growth-factor.html. Accessed March 9, 2011.

[5] Keedy VL, Temin S, Somerfield MR, Beasley MB, Johnson DH, et al.. American Society of Clinical Oncology Provisional Clinical Opinion: Epidermal Growth Factor Receptor (*EGFR*) Mutation Testing for Patients With Non-Small-Cell Lung Cancer Considering First-Line EGFR Tyrosine Kinase Inhibitor Therapy, J Clin Oncol 29:2121-2127, 2011.

[6] Roche, November 23, 2010. http://www.roche.com/media/media_releases/med-cor-2010-11-23.htm.  Accessed November 30, 2010.

[7] Gridelli C, Ardizzoni A, Douillard JY, Hanna N, Manegold C., et al. Recent issues in first-line treatment of advanced non-small-cell lung cancer: Results of an International Expert Panel Meeting of the Italian Association of Thoracic Oncology.  Lung Cancer 68:319-331, 2010. 

[8] Mujoomdar M, Moulton K, Spry C.  Epidermal Growth Factors Receptor Mutation Analysis in Advanced Non-Small Cell Lung Cancer: A Review of the Clinical Effectiveness and Guidelines. Ottawa: Canadian Agency for Drugs and Technologies in Health, 2010.  

[9] NCCN Guidelines Version 1.2011 Updates Non-Small Cell Lung Cancer.

[10] Molina-Vila MA, Bertran-Alamillo J, Reguart N, Taron M, Castella E, Llatjos M, et al.  A sensitive method for detecting EGFR mutations in non-small cell lung cancer samples with few tumor cells.  J Thorac Oncol 3:1224-1235, 2008.

[11] Hoshi K, Takakura H, Mitani Y, Tatsumi K, Momiyama N, Ichikawa Y, et al.  Rapid detection of epidermal growth factor receptor mutations in lung cancer by the Smart Amplification Process.  Clin Cancer Res 13:4974-4983, 2007.

[12] Jackman DM, Miller VA, Cioffredi L et al.  Impact of epidermal growth factor receptor and KRAS mutations on clinical outcomes in previously untreated non-small cell lung cancer patients; results of an online tumor registry of clinical trials.  Clin Cancer Res 15:5267-73, 2009.

[13] European Society of Medical Oncology (ESMO), 2010. Erlotinib improves progression-free survival as first-line therapy in advanced lung cancer - Phase-III data presented at 35th ESMO Congress

http://www.esmo.org/no_cache/view-news.html?tx_ttnews[tt_news]=931&tx_ttnews[backPid]=585bout:Tabs. Accessed December 16, 2010. 

[14] Rosell R, Gervais A, Vergrenegre B, Massati E, Felip F, et al.  Erlotinib versus chemotherapy in advanced non-small-cell lung cancer (NSCLC) patients with epidermal growth factor receptor (*EGFR*) mutations: Interim results of the European Erlotinib versus Chemotherapy (EURTAC) Phase III randomized trial.  ASCO Annual Meeting, June 2011, Chicago, IL.  

[15] Jemal A, Siegel R, Xu J, Ward E.  Cancer Statistics, 2010. CA Cancer J Clin 60:277-300, 2010.

[16] Shepherd FA, Rodrigues Pereira J, Ciuleanu T, et al.  Erlotinib in previously treated non-small-cell lung cancer.  N Engl J Med 353:123-132, 2005.  
